# To what extent did households in developing countries forgo needed healthcare during the COVID-19 pandemic? Repeated survey estimates from 25 countries in 2020 and 2021

**DOI:** 10.1136/bmjph-2024-001027

**Published:** 2024-12-22

**Authors:** Jakub Kakietek, Julia Dayton Eberwein, Amanda Kerr, Nicholas Stacey

**Affiliations:** 1 Health, Nutrition, and Population Global Practice, World Bank Group, Washington, District of Columbia, USA; 2 Association of American Railroads, Washington, District of Columbia, USA; 3 Program in Health Services and Systems Research, Duke-NUS Medical School, Singapore

**Keywords:** COVID-19, Public Health, Cross-Sectional Studies, economics

## Abstract

**Introduction:**

During the first year of the COVID-19 pandemic, health system disruptions, fear of becoming infected with COVID-19, mobility restrictions and lockdowns, and reduced household incomes likely contributed to households forgoing needed healthcare. The objective of this study was to measure the prevalence of forgone healthcare and how its drivers changed between the early period of the pandemic in 2020 and the first half of 2021.

**Methods:**

Using repeated measures collected over two time periods in 63 000 households in 25 low-income and middle-income countries, this analysis documents how the prevalence of forgone healthcare and its drivers changed between 2020 and 2021.

**Results:**

In 2020, in the sample pooled across all countries, 17.9% of households reported not being able to obtain needed healthcare. The prevalence of forgone care was 15.6% in low-income countries (LICs), 17.0% in lower-middle-income countries (LMICs) and 20.5% in upper-middle-income countries (UMICs). In 2021, the prevalence of forgone care was lower—10.3% in the pooled sample, 7.9% in LICs, 15.1% in LMICs and 5.3% in UMICs. Financial barriers were the most common reason for not obtaining needed healthcare in both time periods: 42% in 2020 and 45% in 2021 and were higher in LICs and LMICs than in UMICs.

**Conclusion:**

This study is a comprehensive analysis of the changes in forgone care in low-income and middle-income countries. It documents the predominance of financial barriers among those who could not obtain needed healthcare. It suggests the importance of pandemic preparedness to protect access by vulnerable households to essential healthcare service. The study also demonstrates that phone surveys could, at scale, be a cost-effective way to improve the monitoring of progress towards universal health coverage.

**WHAT IS ALREADY KNOWN ON THIS TOPIC**

- Forgone or delayed healthcare was likely a substantial contributor to excess death during the COVID-19 pandemic, and there is published evidence of substantial forgone care in high-income countries.
- In low-income and middle-income countries, there was some evidence of declines in utilisation, but no cross-country population-based estimates of forgone healthcare and its reasons have been published to date.

**WHAT THIS STUDY ADDS**

- This study documents substantial levels of forgone healthcare in 2020 in a large sample of low-income and middle-income countries, and although forgone care declined in 2021, an important share of the population was still not able to access needed care.
- Overwhelmingly, the main reason that households reported for not obtaining needed healthcare was financial—not having enough money or transportation to obtain healthcare—rather than fear of COVID-19 or lack of staff or supplies in medical facilities.

**HOW THIS STUDY MIGHT AFFECT RESEARCH, PRACTICE OR POLICY**

- The results suggest that mechanisms developed to strengthen pandemic preparedness and resilience of health systems need to be comprehensive, multisectoral and intentionally centred on ensuring that the poorest and most vulnerable households have continuous access to essential health services.
- This study demonstrates that phone surveys with simple and standardised measures could, at scale, be a cost-effective way to improve the measurement and reporting of forgone care and financial protection for better monitoring of progress towards universal health coverage.

## Introduction

 Between 1 January 2020 and 31 December 2021, about 5.4 million COVID-19 deaths were recorded worldwide.^1^ However, recent estimates of excess mortality suggest that between 14.9 and 18.2 million excess deaths could be attributed to the pandemic during this period.^1 2^ Forgone or delayed care—due to health system disruptions (facility closure, insufficient staff, and cancellation of elective procedures), fear of becoming infected with COVID-19, mobility restrictions and lockdowns, reduced household incomes and other reasons—is likely a substantial contributor to this burden of excess deaths.

While quantitative estimates of forgone care during the COVID-19 pandemic have been published for high-income countries (HICs),^3^,^9^ estimates for lower-income countries are largely absent from the literature. Studies based on interviews with health providers and administrative data reported service disruptions during the pandemic.^10^,^14^ A review of the literature on disruptions of family planning and reproductive health services in low-income and middle-income countries found evidence of increased demand, lower utilisation and increased barriers.^15^ However, these studies did not provide insights on whether the declines in utilisation were due to lower demand for services during the pandemic or forgone care, nor do they capture the reasons behind forgoing needed care.

This study aims at filling the gap in our understanding of forgone care in developing countries. In our earlier work, we provided a systematic cross-country analysis of prevalence and reasons behind forgone healthcare in the early period of the pandemic (March–June 2020).^16^ This paper extends that analysis using repeated measures collected with a standardised instrument over two time periods in 25 countries and over 63 000 households, offering a unique insight into the changes in prevalence and drivers of forgone care that occurred between 2020 and the first half of 2021.

## Methods

### Data

This study used data from high-frequency phone surveys (HFPS) of households.^17^ The HFPS initiative was launched by the World Bank in 2020 to monitor the broad socioeconomic impact of COVID-19 on households. The surveys collected information on a variety of topics, including knowledge and concerns about COVID-19; access to food, healthcare, education and social safety nets; changes in employment and income loss; and coping strategies.^18^

Our study presents an analysis of two rounds of HFPS data—one from 2020 and one from 2021—from each of the countries. We included all 25 countries that reported health variables in both waves of data. In 9 of the 25 countries, the sampling frame was drawn from pre-existing nationally representative household surveys. In 13 countries, random digit dialling (RDD) was used; and in three countries, samples were selected based on data obtained from phone operators. In all surveys, information was collected from one respondent per household. In the case of countries where the sampling frame was derived from a previous, in-person survey, this was typically the household head. For surveys whose samples were based on RDD or numbers provided by phone operators, a random adult household member was interviewed. Data collection was typically carried out by national statistics offices. (See online supplemental table S1 for a description of the surveys included in the sample and the World Bank Microdata Library for a detailed description of the collection process for each survey.)

The final sample included 86 643 observations collected from 63 348 households across two waves of data (table 1). Households that were missing data on needed healthcare were omitted from the final sample. This consisted of less than 3% of the observations in 2020 and less than 0.1% in 2021. For 23 295 households, data were collected in both 2020 and 2021 (46,590 observations). These data were analysed as a panel. The remaining 40 053 households were only observed in one survey round. For those countries and households, the sample was a repeated cross-section.

**Table 1:** Sample characteristics

Country income group	Household observations		Country surveys
	2020	2021	
LICs	13 013 (27.0%)	10 282 (26.7%)	5 (20%)
LMICs	23 328 (48.4%)	13 820 (35.9%)	9 (36%)
UMICs	11 819 (24.5%)	14 381 (37.4%)	11 (44%)
Full sample	48 160	38 483	25

Source: authors’ calculations based on high-frequency phone surveys fielded between May 2020 and July 2021.

LICs, low-income countries; LMICs, lower-middle-income countries; UMICs, upper-middle-income countries

### Outcome measures

Respondents (one per household) were asked whether any member of their household needed medical care during the recall period (usually 30 days) and, if so, whether they were able to obtain it. Households were considered to have forgone care when they reported that a household member needed and could not access care. (See online supplemental table S2 for the wording of the health questions and answer options.) Prevalence of forgone care was calculated as the proportion of households that reported a household member not being able to access needed care among households reporting that a household member needed healthcare.

In 21 of the countries, respondents who reported not being able to obtain needed care were asked the main reason. This question was open-ended, and survey enumerators categorised the answers using predefined categories. For this analysis, we grouped the reasons into four categories: (1) financial constraints, which included lack of money, lack of health insurance and lack of transportation; (2) COVID-19-specific reasons, which included fear of COVID-19 and lockdowns or movement restrictions, and stay-at-home orders; (3) reasons pertaining to health service supply constraints, which included unavailability of medical staff or appointments, medicine and supply stockouts, health facility closures and facilities restricting treatment to COVID-19 or emergency cases; and (4) all other reasons not included in the above categories. The rationale for the groupings was to separate reasons attributed directly to the pandemic from more distal ones, where attribution to COVID-19 was less clear. The groupings also helped separate demand-side and supply-side-related reasons.

To put the survey data in a broader context, we also collected key contextual variables: the 7-day average of new COVID-19 cases,^19^ country policy response Oxford stringency index,^20^ percentage of population that received at least one dose of the COVID-19 vaccine^21^ and Google mobility reports.^22^ For each country, the data were extracted for the month preceding survey data collection. Country-level out-of-pocket expenditure data, as a percentage of total health expenditure, were extracted from the Global Health Expenditure Database (GHED) for the most recent year when the data were available for each country in the sample.^23^ Annual changes in gross domestic product (GDP growth) in the two years covered by the studywere extracted from the World Bank’s World Development Indicators database.^24^

### Analysis

This descriptive analysis focused on (1) documenting the extent of forgone care, reasons for forgoing care and differences in those indicators among countries from different income groups; and (2) examining how forgone care and reasons changed between 2020 and 2021.

Household-level data from the 25 countries were pooled into a single data set. Two types of weights were applied. To correct for bias resulting from non-random ownership of phones, sampling weights were developed to adjust for the likelihood of the respondent household owning a phone. To account for the substantial differences in the population of countries included in the sample, household sampling weights were adjusted by the country’s population as a proportion of the population of all countries included in the study for the pooled sample and countries in each income group included in the study for the country income group sample. In this way, each country’s contribution to the average was proportional to its population. We used the same calculated population-weighted averages for the contextual factors. Out-of-pocket expenditure and GDP growth were reported as unweighted averages.

Point estimates were reported for the pooled sample and by country income group. Two sample proportion tests were used to compare the difference between the two time periods and differences between country income groups. For the subset of the surveys that used a panel design, we calculated the proportion of households that reported needing and forgoing care in both time periods. The Strengthening the Reporting of Observational Studies in Epidemiology cross-sectional checklist was used in writing this report.^25^

## Results

### Contextual factors

COVID-19 policy responses became much less stringent in 2021 compared with 2020, with the Oxford stringency index declining and the Google mobility index increasing between the two time periods (online supplemental figure S1). During the same period, the COVID-19 burden increased substantially. On average across countries in the sample, 5.1% of the population had been vaccinated against COVID-19 prior to the 2021 survey, ranging from 0.3% in LICs to 1.4% in lower-middle-income countries (LMICs) and 13.5% in upper-middle-income countries (UMICs).

Out-of-pocket health expenditure accounted for about 38% of total health spending in the full sample, with the lowest proportion in UMICs (32%), higher in LICs (36%) and highest in LMICs (45%) (online supplemental figure S2). The average economy in the full sample contracted by 5.0% in 2020 with growth resuming in 2021. The greatest declines in 2020 took place in the UMIC countries (8.4%); they were smaller in LMICs (4.5%), and lowest in LICs (1.6%). Economic growth resumed in 2021 and was highest in UMICs (8.2%), lower in LMICs (5.9%), and lowest in LICs (3.4%).

### Prevalence of forgone healthcare

**Figure 1:**
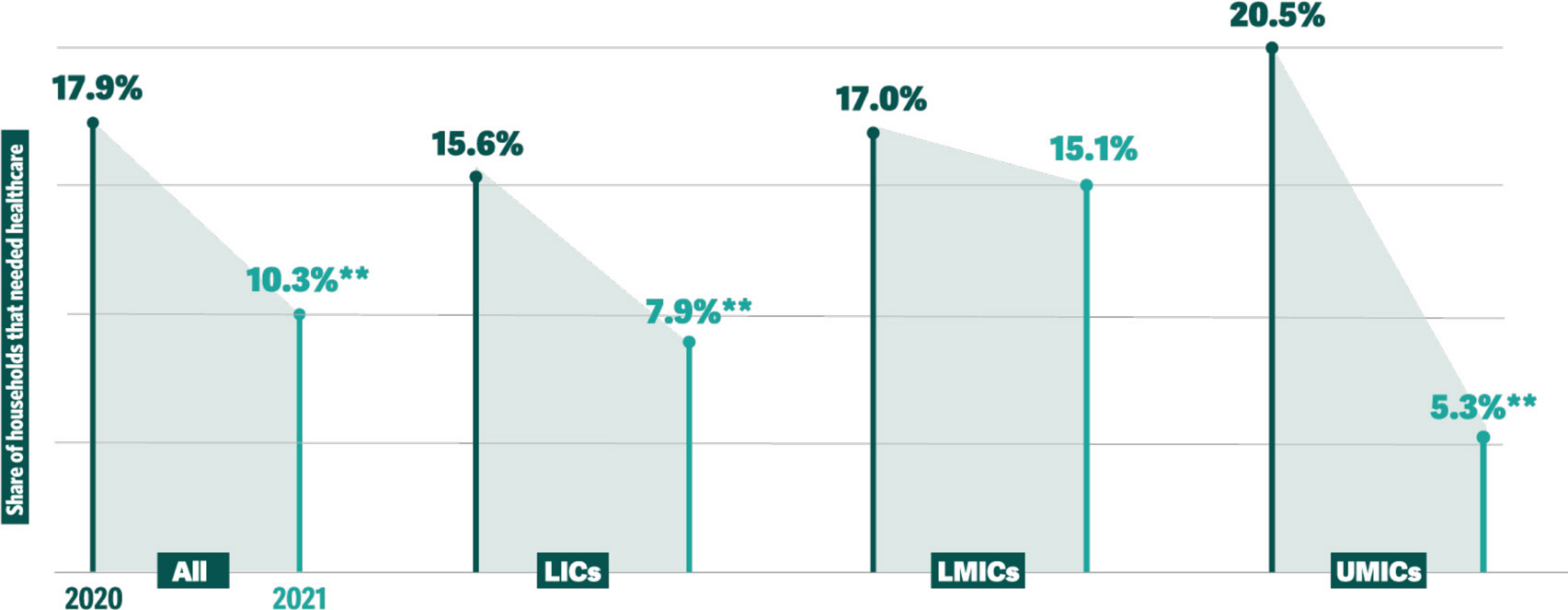
Percentage of households that did not access needed care as share of all households that needed care (forgone care), 2020 and 2021. **p<0.01. LIC, low-income countries; LMIC, lower-middle-income countries; UMIC, upper-middle-income countries.

In 2020, 17.9% (95% CI 16.5% to 19.2%) of households in the pooled sample reported not being able to obtain healthcare when needed, with 15.6% (12.8% to 18.4%) of households reporting forgoing care in LICs, 17.0% (14.8% to 19.3%) in LMICs, and 20.5% (18.5% to 22.5%) in UMICs (figure 1 and online supplemental table S3). In 2021, the prevalence of forgone care in the pooled samples was 10.3% (9.2% to 11.4%) and significantly lower than in 2020 (10.3% vs 17.9%, p<0.01). The prevalence of forgone care was 7.9% (5.8% to 9.9%) in LICs (a 7.8 percentage point decline from 2020, p<0.01), 15.1% (13.0% to 17.2%) in LMICs (a 2.0 percentage point non-significant decline, p=0.21) and 5.3% (4.5% to 6.0%) in UMICs (a 15.3 percentage point decline, p<0.01) (table 2).

**Table 2:** Changes over time (2020–2021) in forgone care and reasons behind forgone care, pooled sample and by country income group

Country group	2020	2021	Diff (PP)
**Forgone care**			
Pooled sample (%)	17.9	10.3	−7.6**
LICs (%)	15.6	7.9	−7.8**
LMICs (%)	17.0	15.1	−2.0
UMICs (%)	20.5	5.3	−15.3**
**Reasons for forgoing care**			
Financial reasons			
Pooled sample (%)	42.0	45.1	3.1
LICs (%)	58.4	41.2	−17.2
LMICs (%)	59.2	72.6	13.4*
UMICs (%)	14.9	20.7	5.8
COVID-19-related reasons			
Pooled sample (%)	17.3	6.4	−10.9**
LICs (%)	5.0	3.8	−1.2
LMICs (%)	17.9	10.0	−7.9**
UMICs (%)	24.6	4.6	−20.0**
Healthcare supply constraints			
Pooled sample (%)	30.7	39.9	9.2*
LICs (%)	31.9	44.7	12.8
LMICs (%)	12.3	9.5	−2.8
UMICs (%)	48.0	66.4	18.5**
Other reasons			
Pooled sample (%)	10.0	7.8	−2.2
LICs (%)	5.0	5.8	0.8
LMICs (%)	10.5	8.0	−2.5
UMICs (%)	12.5	8.9	−3.6

Source: authors’ calculations.

Notes: data are from high-frequency phone surveys fielded between May 2020 and July 2021. Sample is restricted to households reporting some healthcare need during the survey’s recall period. The prevalence of forgone care is the proportion of households that report needing care but not accessing needed care. Financial reasons include lack of money and lack of transportation. COVID-19-related reasons include fear of COVID-19 and movement restrictions. Supply reasons include lack of medical personnel, lack of supplies/medication and facility being closed/full.

Large sample *z*-tests of proportion equality between each income group combination are indicated by stars, with *p<0.05, **p<0.01.

LICs, low-income countries; LMICs, lower-middle-income countries; PP, percentage point; UMICs, upper-middle-income countries

### Reasons behind forgone healthcare

**Figure 2:**
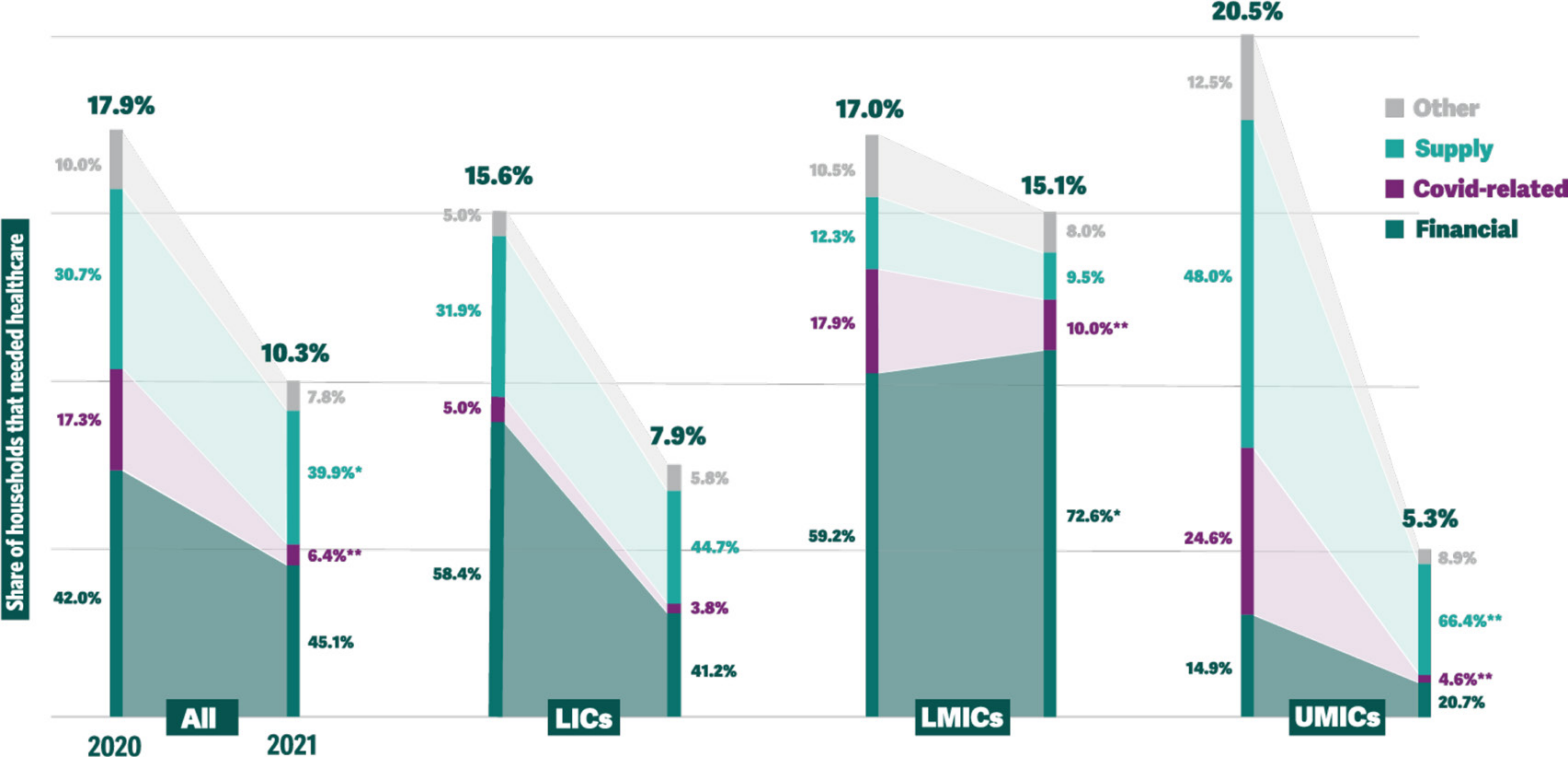
Reasons for forgone care (as a share of households that needed care), 2020 and 2021. *p<0.05; **p<0.01. LIC, low-income countries; LMIC, lower-middle-income countries; UMIC, upper-middle-income countries.

In 2020, in the pooled sample, 42.0% (37.1% to 46.9%) of households reporting forgoing care stated that it was due to financial reasons; 30.7% (26.5 to 35.0) that it was due to lack of supply of health services (unavailability of medical staff and appointments, shortage of supplies/tests, unavailability of medication/drugs, facilities only treating emergencies or COVID-19 cases, long waiting periods); and 17.3% (14.4% to 20.3%) reported that it was due to reasons related directly to COVID-19 (fear of COVID-19, lockdowns and movement restrictions) (figure 2, table 2, and online supplemental table S2). Financial reasons were more commonly reported in LICs than in UMICs (58.4% vs 14.9%, p<0.01) and in LMICs than in UMICs (59.2% vs 14.9%, p<0.01) (table 3). Reasons related to supply constraints were more commonly reported by households in UMICs as compared with those in LICs (48.0% vs 31.9%, p<0.01) and LMICs (48.0% vs 12.3%, p<0.01). Households in LICs were more likely to report supply constraints than households in LMICs (31.9% vs 12.3%, p<0.01). Reasons directly related to COVID-19 were reported more frequently in UMICs than in LICs (24.6% vs 5.0%, p<0.01), and in LMICs compared with LICs (17.9% vs 5.0%, p<0.01).

**Table 3:** Differences across country income groups in forgone care and the reasons, 2020–2021

	Prevalence (per cent)				Difference (percentage point)		
	All countries	LICs	LMICs	UMICs	LIC versus LMIC	LIC versus UMIC	LMIC versus UMIC
**2020**							
Forgone care	17.9	15.6	17.0	20.5	1.4	4.9**	3.5*
Reason for forgone care							
Financial barriers	42.0	58.4	59.2	14.9	0.8	−43.5**	−44.3**
COVID-19	17.3	5.0	17.9	24.6	12.8**	19.6**	6.8
Supply constraints	30.7	31.9	12.3	48.0	−19.6**	16.0*	35.7**
Other	10.0	5.0	10.5	12.5	5.5	7.5*	2.0
**2021**							
Forgone care	10.3	7.9	15.1	5.3	7.2**	−2.6*	−9.8**
Reason for forgone care							
Financial barriers	45.1	41.2	72.6	20.7	31.4**	−20.5*	−51.8**
COVID-19	6.4	3.8	10	4.6	6.2	0.9	−5.3**
Supply constraints	39.9	44.7	9.5	66.4	−35.2**	21.7*	56.8**
Other	7.8	5.8	8	8.9	2.2	3.1	0.9

Source: authors’ calculations.

Notes: data are from high-frequency phone surveys fielded between May 2020 and July of 2021. Sample is restricted to households reporting some healthcare need during the survey’s recall period. The prevalence of forgone care is the proportion of households that report needing care but not accessing needed care. Financial reasons include lack of money and lack of transportation. COVID-19-related reasons include fear of COVID-19 and movement restrictions. Supply reasons include lack of medical personnel, lack of supplies/medication and facility being closed/full.

Large sample *z*-tests of proportion equality between 2020 and 2021 are indicated by stars, with *p<0.05, **p<0.01.

LIC, low-income country; LMIC, lower-middle-income country; UMIC, upper-middle-income country

In 2021, financial constraints were still the most reported reason for forgoing care. In the pooled sample, 45.1% (40.4% to 49.8%) of households reported forgoing care due to financial reasons—a 3.1 percentage point difference that was not statistically significant. Compared with 2020, in 2021 a higher proportion of households reported forgoing care due to reasons related to the supply of services (39.9% vs 30.7%, p=0.01). In contrast, a substantially lower proportion of respondents reported forgoing care due to reasons directly related to COVID-19 (6.4% vs 17.3% in 2020, p<0.01).

The frequency of reporting financial reasons behind forgone care remained high in LICs (41.2% vs 58.4%, p=0.37) and LMICs (72.6% vs 59.2%, p=0.02). As in 2020, financial reasons behind forgone care were reported less frequently in UMICs (20.7% vs 14.9%, p=0.07); an increase of 5.8 percentage points.

The percentage of households reporting forgoing care for reasons related to supply constraints remained similar in LICs and LMICs but increased in UMICs: 44.7% in LICs (an increase of 12.8 percentage points compared with 2020, p=0.28); 9.5% in LMICs (a decrease of 2.8 percentage points, p=0.50); and 66.4% in UMICs (an increase of 18.5 percentage points, p<0.01). The difference between 2020 and 2021 was statistically significant only in UMICs.

In contrast, the proportion of households reporting forgoing care due to reasons directly related to COVID-19 declined significantly in LMICs (10.0% vs 17.9% in 2020, p<0.01) and UMICs (4.6% vs 24.6%, p<0.01), but remained low in LICs (3.8% vs 5.0%, p=0.70).

### Subsample of households with repeated measures

As noted above, repeated measures were available for 23 129 households in 21 countries. In this subsample, 13.9% households reported they could not access needed care in 2020, and 8.2% in 2021 (online supplemental table S4 and figure S3).

Among the households that reported forgoing care in 2020, only 7.8% reported that they could not access care again in 2021; 43.0% of the households that reported forgoing care in 2020 and needing care again in 2021 reported that they were able to access care in 2021; and 49.1% of the households that reported forgoing care in 2020 reported that they did not need care in 2021.

Among the small group of households that reported not being able to access care they needed both in 2020 and 2021, over half said that it was because of financial reasons in both time periods (55.4% in 2020 and 51.9% in 2021). Consistent with the trend in the overall sample, the percentage of households in this small group that reported they could not access services due to fear of COVID-19, movement restrictions or other pandemic containment policies declined from 25.3% in 2020 to 14.8% in 2021, and the percentage reporting forgoing care due to supply-side challenges increased from 15.7% in 2020 to 23.5% in 2021.

## Discussion

This study is the most comprehensive analysis to date of trends in forgone healthcare in low-income and middle-income countries during the COVID-19 pandemic. It shows that the prevalence of forgone care declined from 17.9% in 2020 to 10.3% in 2021. The reductions in forgone care were smallest in LMICs and largest in UMICs. The declines were most likely due to two factors: the loosening of restrictive policy measures and the beginning of the rollout of COVID-19 vaccines in 2021. Indeed, the percentage of respondents reporting forgoing care due to COVID-19-related reasons declined, and declines were largest among UMICs where forgoing care due to fear of getting COVID-19 or lockdown measures was reported by 24.6% of respondents in 2020 but by only 4.6% of respondents in 2021. The declines coincided with: reductions in the COVID-19 Stringency Index, which dropped from 83 in 2020 to 57 in 2021 in the pooled sample and from 91 in 2020 to 61 in 2021 in UMICs; with increases in mobility, with Google Mobility Index rising in the overall sample from 62 to 88, and form 50 to 92 in UMICS; and with the rollout of COVID-19 vaccines, which was faster in UMICs (13.5% of the population was vaccinated with at least one dose of a vaccine in UMICs, 1.4% in LMICs and only 0.3% in LICs included in the sample).

Declines in forgone care were also accompanied by substantial increases in COVID-19 incidence. This suggests that fear of COVID-19 declined despite increases in COVID-19 cases, perhaps indicating that populations became more used to the disease itself, especially as more treatment options became available and with the discovery and rollout of vaccines. However, the extant literature does not provide a robust basis to confirm this hypothesis, and changes in the fear of COVID-19 during the pandemic have not been documented in developing countries. The limited evidence from other regions is inconclusive, with one study from North America and Europe showing increases between March and April 2020 and then declines between April and August 2020, whereas a study of working adults from Japan showing increases in the fear of COVID-19 during the same period.^26 27^

While the prevalence of forgone care declined during the timeframe of our study, financial constraints remained the most reported reason behind it. The percentage of households forgoing care due to financial reasons remained virtually unchanged in the overall sample. This finding is consistent with a large body of literature documenting persistent financial barriers to accessing care in low-income and middle-income countries. However, published studies did not provide information about whether households that reported financial barriers were able to obtain care (ie, by borrowing or selling assets, limiting non-health consumption) or had to forgo healthcare they needed.

In our study, financial barriers were more commonly reported in both time periods in LICs and LMICs, compared with UMICs. This may be because of the overall higher income levels but also likely because prepayments and financial protection mechanisms are better developed in UMICs.^28^ Indeed, out-of-pocket expenditure as a percentage of overall health expenditure was the lowest in UMICs included in the study.^23^ This inequitable impact of financial barriers on forgone care is particularly worrying given the uneven economic recovery from the pandemic. As COVID-19 has subsided, richer countries have been able to recover at a faster pace than poorer ones.^29^ Although the economic contraction was the deepest in the UMICs included in the study (where GDP declined by 8.4%, on average in 2020), the recovery in UMICs was also the strongest (with GDP growth of 8.2% in 2021, on average, compared with 5.9% in LMICs and 3.4% in LICs).

Data on trends in forgone care during the pandemic are extremely limited. As of the writing of this manuscript, no published studies have reported trends in forgone care in developing countries. One study examined trends in the USA and found that the prevalence of reported forgone care declined between August and December 2020 by more than a third (from 41.3% to 27.8%, respectively).^30^ Another US study of a large sample of Medicare beneficiaries showed the overall rate of forgone healthcare was the highest in the summer of 2020 (20.8%), declining in the fall of 2020 (7.8%), and declining further in the winter of 2021 (6.5%).^31^ Our findings are consistent with the declines in reported forgone care in both studies.

Overall, the literature on forgone care in low-income and middle-income countries is limited, with only one published study comparing the levels of forgone care across regions or income groups. That study compared forgone care for non-communicable disease due to financial reasons in 18 countries, spanning LIC, middle-income and HICs and found that forgoing care due to financial reasons was more common in poorer countries, consistent with our findings.^32^ Two regional studies—on from Africa in 2008–2009 and another from the Americas from 2008 to 2018—showed higher average prevalence of forgone care than reported in this study, but the measurement and recall period used were not comparable.^33 34^ Single-country studies from Africa and Asia have reported prevalence of forgone care, using different methodological approaches (eg, vignettes, approaches based on reported health expenditure), also making cross-country comparisons challenging.^35^,^37^

Even fewer published studies examine overtime changes in forgone care in low-income and middle-income countries. One study reported changes in forgone care in Thailand between 2011 and 2019.^38^ The prevalence of forgone care was low—below 3% in the study sample, which is consistent with our data for countries from the East Asia and the Pacific region. The authors argued that this was mostly due to relatively well-developed financial protection mechanisms, which is supported by only a negligible percentage of respondents reporting forgoing care because of financial reasons.

Our analysis has focused on trends in forgone care at the country level to shed light on heterogeneity in trends and drivers of forgone care among different country income groups. In addition, the data also provided a unique opportunity to examine the changes in forgone care overtime at the household level. Among the subset of households with repeated measures, only a very small proportion—less than 10%—of households that reported forgoing care in 2020 also reported forgoing care in 2021. This suggests that forgone care was not symptomatic of chronic lack of access to health services. Over 40% of the households that reported not being able to access services in 2020 and that needed health services in 2021 were able to access them. This is likely due to the overall declining prevalence of forgone care, thanks to improvements in the epidemiological situation and lifting of policy restrictions. Our data show that more than half of the households in the small group experiencing chronic lack of access to needed health services were forgoing care due to financial reasons. Because these results are based on a very small sample of households needing care in both 2020 and 2021, they are only suggestive and point to the need for a thorough overtime study of forgone care.

One limitation of this study is that it relies on data collected by phone, a mode that excludes household without a phone and potentially introducing bias. However, this is unlikely for two reasons. First, cellphone penetration in the countries included in the sample was very high: on average 106 per 100 people in our sample countries had mobile cellular subscriptions.^39^ Second, sampling weights were used to minimise potential selection bias.^40^ Another potential limitation is that forgone care was based on self-reports. Although this is the standard for measuring forgone care, some have argued that alternative methods, such as vignettes or imputations based on reported health expenditure, may be more valid.^35 37^ Such alternative measures are more difficult to administer and require judgement calls from the researchers, and there is no empirical evidence showing that such measures perform better than self-reports. Another limitation is that forgone care was measured at the household level by asking one respondent, often the household head, to provide responses on behalf of the entire household. Although we cannot rule out this potential bias, evidence from an earlier analysis of labour market indicators derived from phone surveys found little evidence of bias from the oversampling of household heads.^41^

In conclusion, this study provides a comprehensive analysis of the changes in forgone care in low-income and middle-income countries during the COVID-19 pandemic. It demonstrates that phone surveys with simple and standardised measures could, at scale, be a cost-effective way to improve the measurement and reporting of forgone care and financial protection for monitoring of the progress towards universal health coverage (UHC), potentially filling a critical data gap.^42 43^ An important next research step will be to replicate the analysis with data from additional countries as well as to continue to monitor trends in forgone care over longer periods of time, generating additional policy use cases.

Although there is strong evidence that phone surveys generate representative results, particularly when based on face-to-face survey sampling frames,^40^ critical next steps in research are needed to continue to improve the quality of the surveys. Specific to health, it will be important to establish standardised measures of forgone care and identify ways to accurately assess healthcare need across cultures and with proxy reporting, as interviewing every household member is expensive and often not feasible. In particular, additional methodological work is needed to achieve representativeness of women.^44^ More generally, where phone surveys samples are based on face-to-face survey sampling frames, it is important to continue periodic face-to-face surveys and make full use of the available information to recalibrate weights for better representativeness. More analysis is also needed to understand the representativeness of phone surveys based on RDD, building on one recent study comparing phone surveys with face-to-face surveys on non-communicable disease risk factors in Colombia found the phone surveys could collect similar health data as household surveys.^45^

While the prevalence of forgone care declined in the overall sample between 2020 and 2021, it was uneven among country income groups, with large declines in LICs and UMICs but little change in LMICs. The analysis shows that even in 2021, large proportions of the population—about 10.8 million households in 2020 and 7.2 million in 2021 in the 25 countries included in the study—could not obtain the healthcare services they needed. In both time periods, almost half of the household who forwent healthcare did so due to financial reason. Surprisingly, factors such as fear of COVID-19 and policy restrictions made relatively small contributions to forgone care, especially in LICs and LMICs. Given the uneven economic recovery from COVID-19 and the global effects of conflicts in Ukraine and the Middle East, it is likely that financial barriers to accessing care will persist and perhaps increase, slowing progress towards achieving UHC. This in turn suggests that comprehensive approaches that address both supply-side and demand-side determinants are needed to preserve the progress made towards achieving UHC. This includes policies that promote inclusive economic growth as well as tax reform to increase revenues for public health spending. At the same time, during times of uncertainty and crisis, it is essential to preserve the most cost-effective basic health services and find ways to remove financial barriers to accessing these essential services.

## supplementary material

10.1136/bmjph-2024-001027 online supplemental file 1

## Data Availability

Data are available in a public, open access repository.
