# Supplementary material for: To what extent did households in developing countries forgo needed healthcare during the COVID-19 pandemic? Repeated survey estimates from 25 countries in 2020 and 2021
Source: BMJ Public Health. 2024 Dec 22;2(2):e001027. doi: 10.1136/bmjph-2024-001027 (PMC11816704; doi:10.1136/bmjph-2024-001027)

Supplementary Table S1: Description and Sample Sizes of Surveys Included in Sample

Country	Sample frame	Panel or repeated cross-section	Income group	Region	2020 Sample		2021 Sample	
					Month	Sample size	Month	Sample size
Argentina	RDD	P	Upper-middle income	Latin America & Caribbean	June	1,001	June	1,216
Bolivia	RDD	P	Lower-middle income	Latin America & Caribbean	June	670	May	1,272
Burkina Faso	Previous survey	P	Low income	Sub-Saharan Africa	June	1,968	April	1,998
Cambodia	Previous survey	CS	Lower-middle income	East Asia & Pacific	May	700	March	378
Colombia	RDD	P	Upper-middle income	Latin America & Caribbean	June	1,000	June	1,221
Costa Rica	RDD	P	Upper-middle income	Latin America & Caribbean	May	801	June	805
Dominican Republic	RDD	P	Upper-middle income	Latin America & Caribbean	May	807	June	1,205
Ecuador	RDD	CS	Upper-middle income	Latin America & Caribbean	June	1,025	May	1,352
El Salvador	RDD	P	Lower-middle income	Latin America & Caribbean	June	804	June	818
Ethiopia	Previous survey	P	Low income	Sub-Saharan Africa	June	3,058	April	1,982
Guatemala	RDD	P	Upper-middle income	Latin America & Caribbean	May	806	June	1,207
Honduras	RDD	P	Lower-middle income	Latin America & Caribbean	June	807	July	1,021
Iraq	Nonsurvey list	CS	Upper-middle income	Middle East & North Africa	August	1,621	June	1,627
Malawi	Previous survey	P	Low income	Sub-Saharan Africa	June	1,729	May	1,540
Mexico	RDD	P	Upper-middle income	Latin America & Caribbean	June	2,109	June	2,625
Mongolia	Previous survey	P	Lower middle income	East Asia & Pacific	May	1,333	June	1,046
Nigeria	Previous survey	P	Lower-middle income	Sub-Saharan Africa	June	1,820	January	1,706
Paraguay	RDD	P	Upper-middle income	Latin America & Caribbean	June	715	June	1,076
Peru	RDD	P	Upper-middle income	Latin America & Caribbean	June	841	June	1,212
Philippines	Nonsurvey list	P	Lower-middle income	East Asia & Pacific	August	9,448	May	2,122
St. Lucia	RDD	CS	Upper-middle income	Latin America & Caribbean	May	1,093	June	835
Sudan	Nonsurvey list	P	Low income	Sub-Saharan Africa	June	4,032	March	2,662
Uganda	Previous survey	P	Low income	Sub-Saharan Africa	June	2,226	March	2,100
Uzbekistan	Previous survey	P	Lower-middle income	Europe & Central Asia	June	1,533	June	1,535
Vietnam	Previous survey	P	Lower-middle income	East Asia & Pacific	June	6,213	March	3,922

Source: Authors' calculations.
Notes: RDD = Random digit dialing; P = Panel; CS = Cross-section.

Supplementary Table S2: Health-related survey questions

**Question***	**Answer options***
1. Have you or any member of your household needed medical treatment during [recall period – typically the past 30 days]?	YES → Question 2<br>NO → Next section
2. Were you or the member of your household able to access the medical treatment?	YES → next section<br>NO → Question 3
3. What was the reason you or the member of your household was not able to access the medical treatment? (single response)	• LACK OF MONEY<br>• LACK OF TRANSPORTATION<br>• NO MEDICAL PERSONNEL AVAILABLE<br>• TURNED AWAY BECAUSE FACILITY WAS FULL<br>• FACILITY IS TOO FAR<br>• SHORTAGES IN MEDICAL SUPPLIES<br>• AFRAID TO VISIT FACILITY DUE TO COVID<br>• FACILITY IS CLOSED<br>• MOVEMENT RESTRICTIONS<br>• OTHER

*This is the most common wording, however there was some variation in word choice across countries. The most common recall period was 30 days but some surveys used different recall periods (for example, “since the beginning of the COVID-19 pandemic,” “since [date of the COVID-19 movement restrictions]” or “in the past 14 days”). **These are the most common answer options, however there was some variation across countries.

Supplementary Figure S1. Changes in Oxford Policy Stringency Index, COVID-19 Incidence, and COVID-19 Vaccination Share

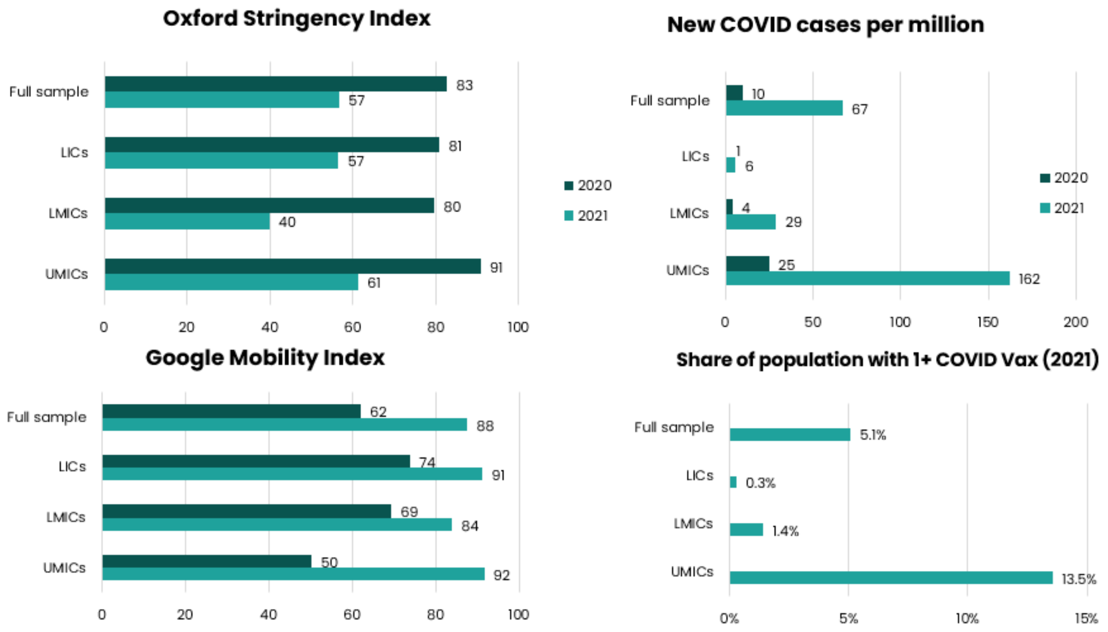

Data sources: Hale et al. 2021 (Oxford Stringency Index); Google 2023 (Google Mobility Index); Dong, Du, and Gardner 2020 (New COVID-19 cases per million); and WHO 2022c (Share of Population with 1+ Vaccination in 2021).
Notes: LICs = Low-income countries, LMICs = Lower-middle-income countries, UMICs = Upper-middle-income countries.

Supplementary Figure S2: Out-of-Pocket Expenditure on Health, as Percentage of Total Current Heath Expenditure and GDP Growth

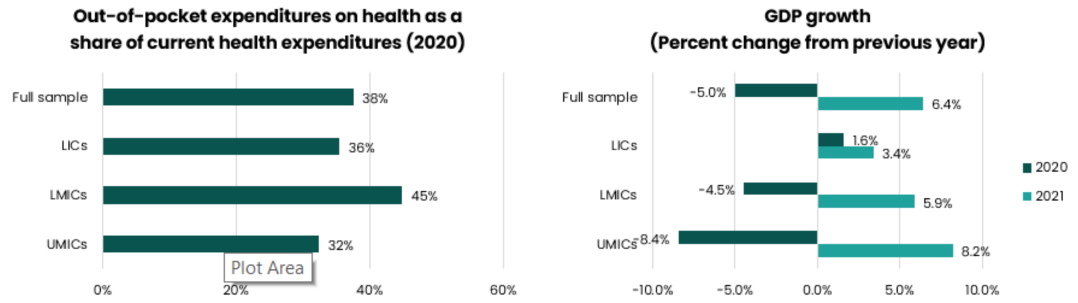

Data sources: WHO 2023 (Out-of-pocket expenditures) and World Bank 2023 (GDP growth).
Notes: LICs = Low-income countries, LMICs = Lower-middle-income countries, UMICs = Upper-middle-income countries.

Supplementary Table S3: Forgone Care and Its Reasons, 2020 and 2021

	Percent of households that did not access care (as share of households that needed health care)		Reasons for forgone care (as share of households not accessing care)							
			(i) Financial		(ii) COVID		(iii) Supply		(iv) Other	
**2020**										
All countries	17.9	[16.5,19.2]	42.0	[37.1,46.9]	17.3	[14.4,20.3]	30.7	[26.5,35.0]	10.0	[6.5,13.5]
LICs	15.6	[12.8,18.4]	58.4	[47.4,69.4]	5.0	[3.3,6.6]	31.9	[21.2,42.6]	5.0	[0.4,9.6]
LMICs	17.0	[14.8,19.3]	59.2	[49.4,68.9]	17.9	[12.6,23.2]	12.3	[6.4,18.2]	10.5	[2.8,18.2]
UMICs	20.5	[18.5,22.5]	14.9	[1,19.9]	24.6	[19.0,30.2]	48.0	[41.4,54.6]	12.5	[8.2,16.8]
**2021**										
All countries	10.3	[9.2,11.4]	45.1	[40.4,49.8]	6.4	[4.3,8.5]	39.9	[34.3,45.5]	7.8	[4.5,11.1]
LICs	7.9	[5.8,9.9]	41.2	[25.4,57.1]	3.8	[-2.3,9.8]	44.7	[24.4,65.0]	5.8	[-5.2,16.8]
LMICs	15.1	[13.0,17.2]	72.6	[66.5,78.7]	10.0	[7.3,12.7]	9.5	[4.2,14.8]	8.0	[5.5,10.5]
UMICs	5.3	[4.5,6.0]	20.7	[16.7,24.8]	4.6	[1.6,7.6]	66.4	[61.5,71.4]	8.9	[4.4,13.3]

Source: Authors' calculations.

Notes: LICs = Low-income countries, LMICs = Lower-middle-income countries, UMICs = Upper-middle-income countries. Data are from High-Frequency Phone Surveys fielded between May and August of 2020. Sample is restricted to households reporting indicating some health care need during survey's recall period. The prevalence of forgone care is the proportion of households that report needing care but not accessing needed care. Financial reasons include lack of money and lack of transportation. COVID-19-related reasons include fear of COVID and movement restrictions. Supply reasons include lack of medical personnel, lack of supplies/medication, or facility being closed/full. Confidence intervals shown in brackets.

Supplemental Table S4. Prevalence and reasons for forgone care in sub-sample of households with repeated measures (N=23,129)

**Panel A**	**N**	**%**
Households with repeated measures	23,129	
Needed care in 2020 as a share of all households with repeated measures	7,542	
Forwent care in 2020 as % of households who needed care in 2020	1,046	13.9%
Needed care in 2021 as a share of all households with repeated measures	8,052	
Forwent care in 2021 as % of households who needed care in 2021	662	8.2%

**Panel B**	**N**	**%**
Forwent care in 2020 and did not need care in 2021 as % of households who forwent care in 2020	514	49.1%
Forwent care in 2020, needed care in 2021, and was able to get care in 2021 as % of households who forwent care in 2020	450	43.0%
Forwent care in 2020 and 2021 as % of households who forwent care in 2020	82	7.8%

**Panel C**	**%**	**%**
Reason for forgoing care as % of households forgoing care in 2020 & 2021	2020	2021
Financial	55.4%	51.9%
COVID-19	25.3%	14.8%
Healthcare supply constraints	15.7%	23.5%
Other	3.6%	9.9%

Notes: Data are from High Frequency Phone Surveys fielded between May 2020 and July of 2021. Financial reasons include lack of money and lack of transportation. COVID-19 related reasons include fear of COVID-19 and movement restrictions. Supply reasons include lack of medical personnel, lack of supplies/medication, and facility closed/full.

Supplemental Figure S3: Prevalence and Reasons for Forgone Care in Subsample of Households with Repeated Measures (*N* = 23,129)

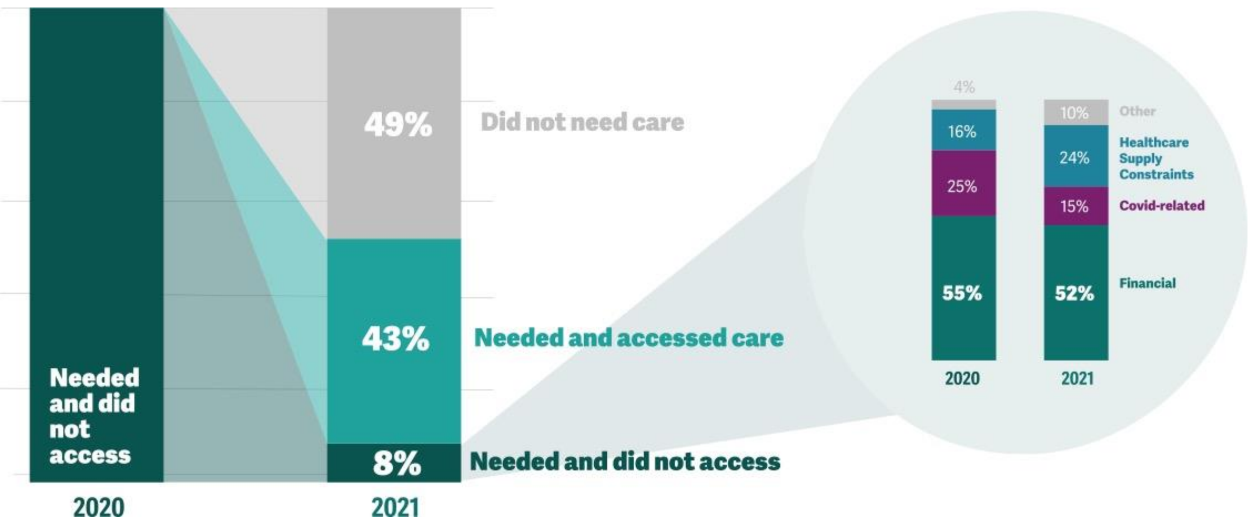

Supplement: online supplemental file 1 [file bmjph-2-2-s001.pdf]
